# Anti-Inflammatory and Anti-Oxidative Activities of Phenolic Compounds from *Alnus sibirica* Stems Fermented by *Lactobacillus plantarum* subsp. *argentoratensis*

**DOI:** 10.3390/molecules22091566

**Published:** 2017-09-18

**Authors:** Thi Tam Le, Jun Yin, MinWon Lee

**Affiliations:** Laboratory of Pharmacognosy and Natural Product based Medicine, College of Pharmacy, Chung-Ang University, Seoul 156-756, Korea; letam18692@gmail.com (T.T.L.); yinjun89@naver.com (J.Y.)

**Keywords:** *Alnus sibirica*, fermentation, diarylheptanoid

## Abstract

Fermentation of *Alnus sibirica* (AS) stems using *Lactobacillus plantarum* subsp. *argentoratensis* was conducted and three compounds isolated from the *Alnus* species were identified for the first time, 7-(3,4-dihydroxyphenyl)-1-(4-hydroxyphenyl)-heptan-3-one, 1-(3,4-dihydroxyphenyl)-7-(4-hydroxyphenyl)-heptan-3-one and 4-(3,4-dihydroxyphenyl)-butan-2-one, along with 14 known compounds. The anti-oxidative and anti-inflammatory abilities of AS and fermented AS (FAS) as well as the isolated phenolic compounds from FAS were investigated. FAS showed stronger anti-oxidative and anti-inflammatory activities than non-fermented AS.

## 1. Introduction

The discovery of new compounds plays a crucial role in drug development. Although there is an extensive diversity of natural compounds, the discovery of novel compounds has become increasingly difficult. Many approaches have been proposed to overcome these challenges. They include exploring unused natural resources from the ocean as well as their secondary metabolites. One of these approaches that has shown promise is fermentation. Along with increasing the overall yield of chemical constituents, fermentation helps to improve pharmacological effects, while also decreasing toxicity [1,2,3,4,5].

The fermentation of traditional medine has been used for a long time in China, Korea and India, etc., and the fermentation of Chinese herbal medicine has been the subject of many investigations because of its undeniable benefits in terms of increasing pharmacological effects with fewer adverse effects [2]. Fermentation is a metabolic process which converts sugars to acids or alcogol, and degrades the organic components through an oxidation-reduction process. The microbial fermentation process is extensively used in the production of food and pharmaceuticals.

*Alnus sibirica* (AS), a member of the *Alnus* species, is geographically distributed throughout Korea, Japan, China and Russia [6]. In Korean traditional medicine, the bark of AS has been used as an antipyretic, an expectorant, antiphlogistils, cough lozenges, an antiasthmatic and a health tea for alcoholism [6]. Together with investigations of the *Alnus* species [7,8], studies on the chemical constituents of AS have led to the isolation of various pharmacogically important compounds. Moreover, we isolated various components from AS including diarylheptanoids, tannins, flavonoids, and triterpenoids [9,10]. In addition, biological activities including anti-oxidative, anti-inflammatory, anti-atopic, melanogenesis inhibitory, anti-adipogenic, anti-tumor, and cytotoxic effects have been studied with the extracts of the plants and the compounds from those plants [10,11,12,13,14,15,16,17,18].

The present paper describes the way the components change through fermentation using *Lactobacillus plantarum* subsp. *argentoratensis* and the enhanced biological activities of Fermented AS (FAS) and the isolated diarylheptanoids from FAS.

## 2. Results and Discussion

### 2.1. Isolation and Structural Identification

**Figure 1:**
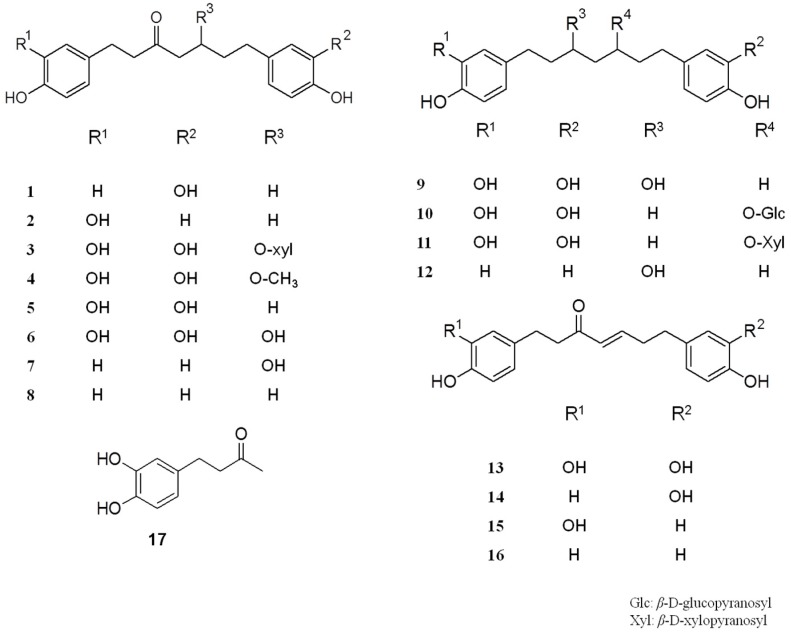
Structures of compounds **1**–**17**.

We successfully isolated 17 compounds from FAS, including three that were novel, from the *Alnus* genus (**1**, **2** and **17**), and 14 other compounds (**3**–**16**). The isolated phenolic compounds were identified as 7-(3,4-dihydroxyphenyl)-1-(4-hydroxyphenyl)-heptan-3-one (**1**), 1-(3,4-dihydroxyphenyl)-7-(4-hydroxyphenyl)-heptan-3-one (**2**), oregonin (**3**) [19], 5-*O*-methylhirsutanonol (**4**) [20], muricarpone B (**5**) [21], hirsutanonol (**6**) [19], 5-hydroxy-3-platyphyllone (**7**) [22], 3-platyphyllone (**8**) [22], rubranol (**9**) [23], rubranoside A (**10**) [24], rubranoside B (**11**) [24], centrolobol (**12**) [25], hirsutenone (**13**) [26], alusenone (**14**) [27], 1-(3,4-dihydroxyphenyl)-7-(4-hydroxyphenyl)-heptene-3-one (**15**) [27], platyphyllenone (**16**) [23], and 4-(3,4-dihydroxyphenyl)-butan-2-one (**17**) [28] (Figure 1).

Compounds **1** and **2** were obtained in a brown syrupy mixture. Positive HR-ESI-MS (high-resolution electrospray ionisation mass spectrometry) revealed two quasi-molecular ion peaks at *m*/*z* 315.1594 ([M + H]^+^, calc. for C_19_H_22_O_4_, 315.1596) and 337.1414 ([M + Na]^+^, calc. for C_19_H_22_O_4_Na, 337.1416). The full carbon and proton signal assignments for **1** and **2** are summarized in Table 1 and Table 2. The ^1^H-NMR spectra of **1** and **2** showed two sets of 1,4-substituted benzene ring signals [δ 6.98 (2H, d, *J* = 8.0, H-2′,6′), 6.71 (2H, d, *J* = 8.0, H-3′,5′) for **1** and 6.96 (2H, d, *J* = 8.0, H-2″,6″), 6.72 (2H, d, *J* = 8.0, H-3″,5″) for **2**], two sets of 1,3,4-trisubstituted benzene ring signals [δ 6.64 (1H, d, *J* = 2.0, H-2″), 6.68 (1H, d, *J* = 8.0, H-5″), 6.47 (1H, dd, *J* = 2.0, 8.0, H-6″) for **1** and 6.65 (1H, d, *J* = 2.0, H-2′), 6.68 (1H, d, *J* = 8.0, H-5′), 6.49 (1H, dd, *J* = 2.0, 8.0, H-6′) for **2**], and six methylenes in the heptane moiety of **1** and **2** [δ 1.45–2.75 (24H, m, H-1,2,4,5,6,7)] that connected with two different aromatic ring systems. The ^13^C-NMR spectra of **1** and **2** included duplicate signals, indicating a close structural similarity between **1** and **2,** suggesting that they likely comprise a pair of isomers with the molecular formula of C_19_H_22_O_4_ (Table 1).

**Figure 2:**
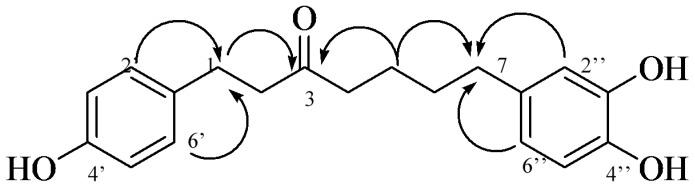
Important HMBC correlations of compound **1**.

Additionally, the HMBC (heteronuclear multiple-bond correlation) correlations of H-2′, 6′ (δ 6.98) to C-1 (δ 28.76) justified the inferred connectivity between C-1′ of 4′-hydroxyphenyl and C-1 of the heptane moiety, while correlations from H-2″ (δ 6.64) and H-6″ (δ 6.47) to C-7 (δ 34.68) revealed the junction between C-1″ of the 3″, 4″-dihydroxyphenyl and C-7 of the heptane moiety in **1**. Thus, the structure of **1** was elucidated as 1-(4-hydroxyphenyl)-7-(3,4-dihydroxyphenyl)-3-heptanone (Figure 2).

**Figure 3:**
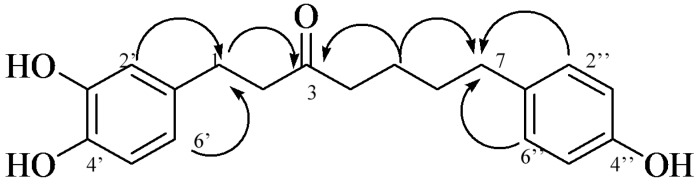
Important HMBC correlations of compound **2**.

We next applied the same methods to **2**, inferring the connectivity between C-1′ of 3′, 4′-dihydroxyphenyl and C-1 of the heptane moiety for **2** from the correlations of H-2′ (δ 6.65) and H-6′ (δ 6.49) with C-1 (δ 28.98), while the junction between C-1″ of the 4″-hydroxyphenyl moiety and C-7 of the heptane moiety were inferred from the correlations between H-2″, 6″ (δ 6.96) and C-7 (δ 34.49). Thus, the structure of **2** was determined to be 1-(3,4-dihydroxyphenyl)-7-(4-hydroxyphenyl)-3-heptanone (Figure 3).

The data are the first descriptions of **1** and **2** from the *Alnus* genus. However, spectroscopic data of **1** and **2** were reported in a study of *Amomum muricarpum* [29].

**Figure 4:**
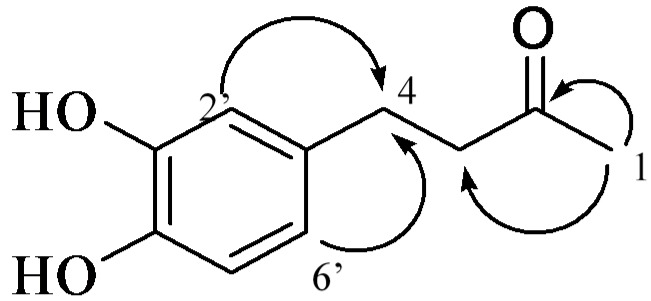
Important HMBC correlations of compound **17**.

Compound **17** was isolated as a black oil. The positive HR-ESI-MS showed two quasi-molecular ion peaks at *m*/*z* 181.0858 ([M + H]^+^, calc. for C_10_H_12_O_3_, 181.0856) and 203.0682 ([M + Na]^+^, calc. for C_10_H_12_O_3_Na, 203.0684). The ^1^H-NMR spectra of **17** indicated the presence of the ABX spin system 6.40 (1H, dd, *J* = 1.9, 8.1 Hz, H-6′), 6.54 (1H, d, *J* = 1.9 Hz, H-2′), and 6.60 (1H, d, *J* = 8.1 Hz, H-5′). The ^13^C-NMR spectra of **17** revealed one carbonyl group, δ 208.4 (C-2), and six aromatic carbons: C-1′ (δ 132.3); C-2′ (δ 115.9); C-3′ (δ 145.4); C-4′ (δ 143.7); C-5′ (δ 116.1); and C-6′ (δ 119.1). The HMBC spectra of **17** displayed a correlation of H-2′ (δ 6.54) and H-6′ (δ 6.40) with C-4 (δ 28.9), as well as a correlation between H-1 (δ 2.05, CH_3_) and the ketone C-2 (δ 208.4) (Figure 4). Together, these data confirmed the structure of **17** as 4-(3,4-dihydroxyphenyl)-butan-2-one. Although **17** has been previously isolated from rat urine after feeding the rats a related compound (5-hydroxy-1,7-bis(3,4-dihydroxyphenyl)-1-hepten-3-one) [30], and although it was also synthesized as an intermediate in the biosynthesis of cyclic phenolic natural products [28], in this research, **17** was isolated from a plant for the first time.

### 2.2. Evaluation of Anti-Oxidative and Anti-Inflammatory Activities

AS, FAS and all the isolated phenolic compounds from FAS were measured to assess their anti-oxidative activities. Both AS and FAS exhibited potent anti-oxidative activities and FAS showed a more potent activity than AS (Table 2). All compounds exhibited potent anti-oxidative activities except **8** (Table 2). Moreover, compounds **3**–**6**, **9**–**13** exhibited more potent radical scavenging activity than ascorbic acid (the positive control). Compounds **3**, **6**, and **13** showed a strong anti-oxidative activity, followed by **1**, **2** and **10,** which is similar to the results in Reference [27]. Moreover, **4** and **5** also showed potent anti-oxidative activity, especially **5**, which was newly produced, and fermentation led to the obviously increased content of **13** [31], and this it had the most potent anti-oxidative activity ability. This may be the key of the reason that FAS showed stronger anti-oxidative activity.

Furthermore, the nitric oxide (NO) production inhibitory effects of AS, FAS and all of the isolated phenolic compounds from FAS in LPS-stimulated RAW 264.7 cells were evaluated. While all samples including AS, FAS and all the isolated compounds showed more potent nitric oxide production inhibitory effects than L-NMMA (positive control), FAS exhibited stronger activity than AS, and **3**, **5**, **9**, **13** showed potent NO production inhibitory activities (Table 3) [32,33].

## 3. Experimental Section

### 3.1. General Experimental Procedure

Column chromatography was performed using Sephadex LH-20 (10–25 μm, GE Healthcare Bio-Science AB, Uppsala, Sweden) and MCI-gel CHP 20P (75–150 μm, Mitsubishi Chemical, Tokyo, Japan). ODS-B gel (40–60 μm, Daiso, Osaka, Japan) provided a stationary phase in the middle-pressure liquid chromatography (MPLC) system (Gilson, Seoul, Korea). For monitoring of each fraction, thin layer chromatography (TLC) was carried out using pre-coated silica gel 60 F_245_ plates (Merck, Darmstadt, Germany) developed with chloroform, methanol, and water (70:30:4 or 80:20:2 volume ratio). Spots were detected under ultraviolet (UV) radiation (254 nm) and by spraying with FeCl_3_ and 10% sulfuric acid (H_2_SO_4_) or anisaldehyde–H_2_SO_4_ followed by heating. Chemical structures were elucidated by combining the data analysis from several instruments: The 1D nuclear magnetic resonance (NMR) such as ^1^H-(300 or 600 MHz) and ^13^C-(150 MHz) NMR, 2D-NMR including proton-proton correlation spectroscopy (^1^H-^1^H-COSY), heteronuclear single quantum coherence (HSQC), heteronuclear multiple bond coherence (HMBC), and FT-IR experiments that were recorded with Gemini 2000 and VNS (Varian, Palo Alto, CA, USA) at the center for research facilities at Chung-Ang University.

### 3.2. Plant Material

The stems of AS were collected from Kuksabong, Seoul, Republic of Korea, in January 2015, certificated by Prof. Min Won Lee (Pharmacognosy Laboratory, College of Pharmacy, Chung-Ang University). A voucher specimen was deposited at the herbarium of the College of Pharmacy, Chung-Ang University (Seoul, Korea).

### 3.3. Extraction and Fermentation

The stems of AS (2.7 kg) were extracted with 80% prethanol A (30 L) at room temperature followed by removal of the prethanol A under vacuum, to yield 312 g of the extract. One hundred grams of the extract was fermented with *Lactobacillus plantarum* subsp. *argentoratensis*.

The *L. plantarum* subsp*. argentoratensis* strain was purchased from the Korean Agricultural Culture Collection. The purchased strain was grown in Lactobacilli MRS broth (6.4% dextrose, 18.2% proteose peptone No. 3, 18.2% beef extract, 9.1% yeast extract, 9.1% sodium acetate, 1.8% polysorbate 80, 3.6% amonium citrate, 3.6% dipotassium phosphate, 0.9% magnesium sulfate, and 0.5% manganese sulfate). The strain was inoculated in liquid media and then incubated for 48 h at 32 °C.

AS extract was sterilized for 20 min at 121 °C after adding 9 L of water. Sterilized AS was then inoculated in a liquid medium, mixed at room temperature, and fermented for seven days. After the fermentation, it was evaporated into the final fermented FAS.

### 3.4. Isolation

The fermented extract (312 g) was partitioned with ethyl acetate (5 L) and water (5 L). The ethyl acetate layer was further processed using Sephadex LH-20 column (10 cm× 120 cm, GE Healthcare Life Sciences, Uppsala, Sweden) and eluted with a water–methanol gradient (from 10:0 to 0:10) and 50% acetone, yielding 10 fractions.

Fraction 2 (1.1 g) was applied to a MCI CHP20P column (5 cm × 60 cm, Supelco Inc, Bellefonte, PA, USA) with a water–methanol gradient (from 2:8 to 0:10) to yield oregonin (**3**, 145 mg) and hirsutenone (**13**, 560 mg). Fraction 3 (2.1 g) was applied to a MCI CHP20P column (5 cm × 60 cm) with a water–methanol gradient (from 5:5 to 0:10) to obtain rubranoside B (**11**, 70 mg). Fraction 4 (1.3 g) was applied to a MCI CHP20P column (5 cm × 60 cm) a gradient solvent system of water–methanol and ODS-B gel column (4 cm × 40 cm, OSAKA SODA CO., LTD., Osaka, Japan) using a water–methanol gradient (from 10:0 to 0:10) in the MPLC system to collect rubranoside A (**10**, 40 mg). Fraction 6 (5.8 g) was applied to a MCI CHP20P column (5 cm × 60 cm) a gradient solvent system of water–methanol (from 5:5 to 0:10) to obtain six sub-fractions and hirsutenone (**13**, 450 mg). Fraction 6-5 (900 mg) was added to an ODS-B gel column (4 cm × 60 cm) using a water–methanol gradient (from 10:0 to 0:10) in the MPLC system to collect 5-hydroxy-3-platyphyllone (**7**, 39 mg) and rubranoside B (**11**, 100 mg). Fraction 7 (2.2 g) was subjected to MCI CHP20P column chromatography (5 cm × 60 cm) using a gradient solvent system of water–methanol (from 6:4 to 0:10) to yield hirsutanonol (**6**, 310 mg) and centrolobol (**12**, 65 mg). Fraction 8 (7.1 g) was applied to a MCI CHP20P column (5 cm × 120 cm) using a gradient solvent system of water–methanol (from 10:0 to 0:10) to obtain eight sub-fractions. Fraction 8-4 (1.2 g) was applied to an ODS-B gel column (5 cm × 60 cm) using the water–methanol gradient (from 10:0 to 0:10) in the MPLC system to collect four sub-fractions. Fraction 8-4-4 ( 300 mg) was further separated by column chromatography on a Sephadex LH-20 column (3 cm × 80 cm) with 80% ethanol to obtain mixture of 7-(3,4-dihydroxyphenyl)-1-(4-hydroxyphenyl)-heptan-3-one (**1**) and 1-(3,4-dihydroxyphenyl)-7-(4-hydroxyphenyl)-heptan-3-one (**2**) (mixture of **1** and **2**, 30 mg), alusenone (**14**) and 1-(3,4-dihydroxyphenyl)-7-(4-hydroxyphenyl)-heptene-3-one (**15**) (mixture of **14** and **15**, 50 mg), platyphyllenone (**16**, 50 mg). Similar to fraction 15-4,5-*O*-methylhirsutanonol (**4**, 70 mg) and 4-(3,4-dihydroxyphenyl)-butan-2-one (**17**, 15 mg) were obtained from fraction 8-2. Fraction 9 (1.3 g) was subjected to MCI CHP20P column (5 cm × 60 cm) a gradient solvent system of water–methanol (from 7:3 to 0:10) and then further with ODS-B gel column (3 cm × 40 cm) using the water–methanol gradient (from 10:0 to 0:10) in the MPLC system, finally, purified with Sephadex LH-20 column (2 cm × 40 cm) with 80% ethanol to collect muricarpone B (**5**, 380 mg), 3-platyphyllone (**8**, 35 mg), rubranol (**9**, 81 mg).

Compounds **1** and **2**, brown syrup, C_19_H_22_O_4_ positive HR-ESI-MS (*m*/*z* 315.1594 ([M + H]^+^, calc. for C_19_H_22_O_4_, 315.1596 and 337.1414 ([M + Na]^+^, calc. for C_19_H_22_O_4_Na, 337.1416). IR v*_max_* cm^‒1^: 1697 (CO), 1611, 1513 (Ar). ^1^H-NMR (DMSO-*d*_6_, 600 MHz) and ^13^C-NMR (DMSO-*d*_6_, 150 MHz): see Table 1.

Compound **17**, black oil, C_10_H_12_O_3_ positive HR-ESI-MS (*m*/*z* 181.0858 ([M + H]^+^), calc. for C_10_H_12_O_3_, 181.0856 and 203.0682 ([M + Na]^+^), calc. for C_10_H_12_O_3_Na, 203.0684). IR v*_max_* cm^‒1^: 1682 (CO), 1604, 1523 (Ar). ^1^H-NMR (DMSO-*d*_6_, 600 MHz) δ 2.05 (3H, s, H-1), 2.6 (4H, m, H-3 and H-4), 6.40 (1H, dd, *J* = 1.9, 8.1 Hz, H-6′), 6.54 (1H, d, *J* = 1.9 Hz, H-2′), 6.60 (1H, d, *J* = 8.1 Hz, H-5′). ^13^C-NMR (DMSO-*d*_6_, 150 MHz) δ 28.9 (C-4), 30.3 (C-1), 45.1 (C-3), 116.0 and 115.9 (C-2′ and C-5′), 132.6 (C-1′), 143.6 (C-4′), 145.2 (C-3′), 208.3 (C-2).

### 3.5. Measurement of DPPH Radical Scavenging Activity

First 20 μL of each sample was added into 180 μL 2,2-diphenyl-1-picrylhydrazyl (DPPH) solution 0.2 mM in absolute ethanol. An ELISA reader (TECAN, Salzburg, Austria) was used to measure the optical density of the solution at 540 nm after mixing and incubating for 30 min at room temperature. DPPH radical scavenging activity was calculated as inhibition rate (%) = [1 − (sample optical density/control optical density)] × 100. The IC_50_ value was defined as the concentration at which 50% of the DPPH free radicals were scavenged. Ascorbic acid was used as a positive control.

### 3.6. Cell Culture

Mouse monocyte-macrophage RAW 264.7 cells (Korean Cell Line Bank) were maintained in DMEM media supplemented with penicillin (100 U/mL) and 10% fetal bovine serum at 37 °C in a humidified incubator with 5% CO_2_ and 95% air, the medium was changed every three days.

### 3.7. Measurement of Inhibition of NO Production

RAW 264.7 macrophages were seeded at density of 1 × 10^5^ cells per well in 200 μL in a 96-well plate. The cells were incubated at 37 °C in 5% CO_2_ for 3 h to ensure adherence. Afterwards, the medium was removed and the cells were treated with AS, FAS (100, 50, 25, 12.5, 6.125 μg/mL, DMEM medium) and isolated phenolic **1**–**17** (100, 50, 25, 12.5, 6.125 μM, DMEM medium) for 1 h. The cells were stimulated with 10 μg/mL lipopolysaccharide (LPS) for 24 h. The spent media were collected and analyzed for nitric oxide (NO) production measurement with Griess reagent by mixing 100 μL of cell culture supernatant with the same volume of reagent. NO production inhibitory activity (I) was calculated as inhibition rate (%) calculated from the following formula. L-N^G^-monomethyl arginine citrate (L-NMMA) was used as the positive control.

$$I=\frac{1-\left(A\;\mathrm{sample}-A\;\mathrm{blank}\right)}{A\;\mathrm{control}-A\;\mathrm{blank}}\times 100$$

where: A sample: Absorbance of compound-treated cell sample; A control: Absorbance of only LPS-treated cell sample; A blank: Absorbance of non-treated cell sample.

### 3.8. Statistical Analysis

All data are expressed as mean±standard deviation (SD). Values were performed by one-way analysis of variance (ANOVA) followed by post hoc Turkey test *p* < 0.05 value was used to determine statistical significance. All analyses were performed at least three independent experiments and each experiment was analyzed at least triplicate. In all experiments, untreated cells were considered as blank (B), treated cells with only LPS were considered as control (C).

## 4. Conclusions

The fermentation of AS stems was conducted and it yielded three phenolic compounds (**1**, **2** and **17**) from the *Alnus* species isolated for the first time, along with 14 known diarylheptanoids (**3**–**16**). Additionally, FAS and its isolated phenolic compounds showed more potent anti-oxidative and anti-inflammatory activities than AS which contributed clear evidence indicating that fermentation is a promising approach to elevate the value from natural resources.

## Figures and Tables

**Table 1:** ^13^C and ^1^H-NMR data of compounds **1** and **2**.

Position	1		Position	2	
	(DMSO-*d*_6_, 600 and 150 MHz)			(DMSO-*d*_6_, 600 and 150 MHz)	
	δ_C_	δ_H_		δ_C_	δ_H_
1	28.76	2.65–2.75 (m)	1	28.98	2.65–2.75 (m)
2	44.08	2.65–2.75 (m)	2	44.12	2.65–2.75 (m)
3	210.72		3	210.72	
4	42.16	2.45–2.55 (m)	4	42.16	2.45–2.55 (m)
5	23.15	1.45–1.55 (m)	5	23.12	1.45–1.55 (m)
6	31.09	1.45–1.55 (m)	6	31.09	1.45–1.55 (m)
7	34.68	2.45–2.55 (m)	7	34.49	2.45–2.55 (m)
1′	131.71		1″	132.45	
2′, 6′	129.67	6.98 (2H, d, *J* = 8.0)	2″, 6″	129.51	6.96 (2H, d, *J* = 8.0)
3′, 5′	115.37	6.71 (2H, d, *J* = 8.0)	3″, 5″	115.32	6.72 (2H, d, *J* = 8.0)
4′	155.53		4″	155.37	
1″	133.39		1′	132.62	
2″	115.25	6.64 (1H, d, *J* = 2.0)	2′	115.28	6.65 (1H, d, *J* = 2.0)
3″	145.03		3′	145.14	
4″	143.22		4′	143.41	
5″	115.74	6.68 (1H, d, *J* = 8.0)	5′	115.96	6.68 (1H, d, *J* = 8.0)
6″	119.32	6.47 (1H, dd, *J* = 2.0, 8.0)	6′	119.21	6.49 (1H, dd, *J* = 2.0, 8.0)

dd: double doublet; *J*: *J*-value.

**Table 2:** IC_50_ values of compounds 1–**17** for DPPH radical scavenging activity.

Samples	IC_50_ (μg/mL)	Compounds	IC_50_ (μM)
**AS**	27.12 ± 3.00 ^c^	**1 + 2**	47.51 ± 1.00 ^d^
**FAS**	12.64 ± 3.61 ^b^	**3**	32.13 ± 0.22 ^b,c^
**Ascorbic acid**	3.04 ± 0.12 ^a^	**4**	28.34 ± 0.35 ^b,c^
		**5**	20.13 ± 0.13 ^a^
		**6**	24.98 ± 0.21 ^a,b^
		**7**	107.79 ± 1.07 ^h^
		**8**	623.41 ± 13.09 ^i^
		**9**	34.87 ± 2.98 ^c^
		**10**	45.11 ± 1.66 ^d^
		**11**	31.07 ± 0.86 ^b,c^
		**12**	44.40 ± 0.96 ^d^
		**13**	28.99 ± 1.07 ^b,c^
		**14 + 15**	60.31 ± 0.39 ^e^
		**16**	101.21 ± 1.03 ^g^
		**17**	68.89 ± 2.00 ^f^
		**Ascorbic acid**	46.03 ± 0.45 ^d^

Values are presented as mean ± SD (*n* = 3). Different superscript letters indicate a significant difference (*p* value < 0.05).

**Table 3:** IC50 values of compounds **1**–**17** for inhibition of nitric oxide production.

Samples	IC_50_ (μg/mL)	Compounds	IC_50_ (μM)
**AS**	12.34 ± 0.44 ^b^	**1 + 2**	15.57 ± 3.24 ^b,c^
**FAS**	4.22 ± 0.39 ^a^	**3**	9.69 ± 2.34 ^a,b^
**L-NMMA**	35.53 ± 3.45 ^c^	**4**	11.45 ± 2.86 ^a,b^
		**5**	6.57 ± 1.18 ^a^
		**6**	21.28 ± 2.93 ^c,d^
		**7**	30.00 ± 2.86 ^e^
		**8**	32.12 ± 3.00 ^e^
		**9**	10.25 ± 2.51 ^a,b^
		**10**	81.91 ± 5.98 ^g^
		**11**	38.90 ± 4.01 ^f^
		**12**	27.77 ± 1.30 ^d,e^
		**13**	5.77 ± 2.35 ^a^
		**14** **+** **15**	11.94 ± 4.03 ^a,b^
		**16**	15.85 ± 2.06 ^b,c^
		**17**	22.15 ± 2.91 ^c,d^
		**L-NMMA**	41.95 ± 6.48 ^f^

Values are presented as mean ± SD (*n* = 3). Different superscript letters indicate a significant difference (*p* value < 0.05).
